# Gender differences in cognitive benefits of meeting physical activity guidelines in older Chinese adults

**DOI:** 10.3389/fpubh.2025.1539369

**Published:** 2025-06-11

**Authors:** Wang Li, Peiyou Chen, Aiyun Jiang, Fan Zhang, Zhijian Wu

**Affiliations:** ^1^School of Sport Sciences, Nanjing Normal University, Nanjing, China; ^2^Department of Basic Courses, Xinjiang Industry Technical College, Ürümqi, China; ^3^School of Special Police, Nanjing Police University, Nanjing, China

**Keywords:** accelerometer, cognitive function, gender differences, older adults, physical activity

## Abstract

**Objective:**

This study investigates the relationship between adherence to WHO physical activity guidelines and cognitive function in older Chinese adults, with a particular focus on gender-specific effects. We hypothesize that meeting physical activity guidelines is positively associated with cognitive function and that the magnitude of this association differs between men and women.

**Methods:**

We conducted a cross-sectional study involving 308 community-dwelling older adults in Nanjing, China (mean age = 68.4 years, SD = 5.6). Participants were stratified by gender and age group (60–70 vs. 71–80 years). Physical activity was objectively measured using ActiGraph GT3X+ accelerometers, and cognitive function was assessed via the Montreal Cognitive Assessment (MoCA). Multivariable linear regression was used to examine associations between physical activity adherence and cognitive function, adjusting for age, education, BMI, and self-rated health. Gender differences were analyzed using ANOVA, and interaction terms were included to assess moderation effects.

**Results:**

Older adults meeting the WHO-recommended 150 min of moderate-to-vigorous physical activity per week had significantly higher MoCA scores than non-adherent individuals (*β* = 3.67, 95% CI: 3.04–4.29, *p* < 0.001; Cohen’s *d* = 0.85). Women showed greater improvements in executive function (*β* = 0.79, *p* = 0.043) and visuospatial abilities (*β* = 0.47, *p* = 0.017), while men demonstrated greater memory gains (*β* = 1.31, *p* < 0.001). A significant interaction between gender and physical activity adherence (*p* = 0.008) suggested distinct cognitive benefits across sexes.

**Conclusion:**

Adherence to physical activity guidelines is associated with cognitive health in older adults, with gender-specific variations in cognitive benefits. These findings underscore the importance of tailored public health interventions that consider gender differences in cognitive aging to optimize cognitive outcomes.

## Introduction

With the growing global aging population, maintaining cognitive function in older adults has become a pressing public health issue. By 2050, the number of individuals aged 60 and above is projected to reach 2.1 billion globally (1). Cognitive decline, closely linked to the rising incidence of dementia, places a substantial economic and psychological burden on society and families (2). Therefore, identifying interventions that can delay or prevent cognitive decline is of critical public health significance.

Physical activity is widely recognized as a modifiable lifestyle factor that provides substantial health benefits for older adults. The World Health Organization (WHO) recommends that adults engage in at least 150 min of moderate-intensity physical activity per week to support overall health (3). Numerous studies have demonstrated that physical activity not only reduces the risk of chronic conditions such as cardiovascular disease, diabetes, and depression (4), but also has a positive impact on cognitive function in older adults (5). For example, a systematic review and meta-analysis by Blondell et al. (6) reported that physical activity is significantly associated with a reduced risk of cognitive decline. Despite this growing body of evidence, the underlying mechanisms, the moderating role of gender, and the cultural specificity of these effects remain insufficiently understood and warrant further investigation.

Recent studies have proposed the cognitive reserve hypothesis, which suggests that engaging in enriching activities, such as physical exercise, enhances brain plasticity and helps buffer against age-related cognitive decline (7). This hypothesis has been widely supported, particularly in explaining how physical activity may protect cognitive function. However, much of the existing literature has focused on Western populations, with relatively few studies investigating the relationship between physical activity and cognitive health among older adults in China. Research from other Asian populations, such as Japan and South Korea, suggests that cultural factors, lifestyle habits, and societal expectations may influence the impact of physical activity on cognitive function differently than in Western contexts (8). A study in Japan, for example, found that socially integrated physical activities such as group exercise classes had a stronger cognitive benefit in women than in men (9). In contrast, research in South Korea indicated that men engaging in structured resistance training showed greater memory improvements compared to women (10). These findings highlight the need to explore how similar or differing trends manifest among Chinese older adults.

Gender differences are an important factor in studies of cognitive aging. Research has shown that men and women follow different trajectories of cognitive aging. For example, Finkel et al. (9) found that women tend to outperform men in verbal fluency and executive function, while men show superior performance in spatial memory and visuospatial abilities (11). These differences may be driven by biological factors (such as hormone levels) and sociocultural influences (such as gender roles) (12). Additionally, men and women differ in their levels of physical activity. Men are more likely to engage in high-intensity physical activities, while women tend to participate in lower-intensity, longer-duration activities (13). However, the moderating role of gender in the relationship between physical activity and cognitive function remains underexplored, particularly among older adults in China.

To address these gaps, this study examines the relationship between adherence to physical activity guidelines and cognitive function in a sample of older adults in China, with a focus on the moderating role of gender. We hypothesize that: Meeting physical activity guidelines is positively associated with cognitive function. Gender moderates this relationship, with men and women experiencing distinct cognitive benefits. Using objective physical activity measurements obtained through accelerometers and a comprehensive cognitive function assessment via the Montreal Cognitive Assessment (MoCA), this study not only provides more precise evidence of the impact of physical activity on cognitive health but also sheds light on the key role gender differences play in this process.

## Methods

### Study design and participants

This cross-sectional study aims to examine the relationship between adherence to physical activity guidelines and cognitive function among older adults in China, with a particular focus on the moderating role of gender. The study was approved by the Ethics Committee of Nanjing Normal University (Approval No: 202003005), and all participants provided written informed consent before participation.

The study participants were community-dwelling older adults aged 60–80 years residing in Nanjing, China. The inclusion criteria were: (a) aged between 60 and 80 years at the time of the survey; (b) no diagnosis of severe psychiatric disorders such as major depression or schizophrenia, as confirmed by clinical records and standardized assessments; (c) ability to communicate effectively, with no reported or diagnosed language impairments; and (d) adequate vision, hearing, and cognitive status to complete all assessments. Participants with significant speech, hearing, or visual impairments, or with severe cognitive or psychiatric conditions that might interfere with participation, were excluded. Detailed screening thresholds (e.g., visual acuity, audiometry, and MMSE cutoffs) are provided in the Supplementary Table S1. To ensure a balanced and representative sample, participants were stratified by age into two groups (60–70 and 71–80 years), with approximately equal numbers of males and females in each group.

The total population of community-dwelling older adults aged 60 and above in Nanjing, China, is approximately 1,600,000 individuals. To detect significant gender-related differences in cognitive functions, a power analysis was conducted using G Power (Version 3.5). Assuming a medium effect size (Cohen’s *d* = 0.5), an alpha level of 0.05, and a desired statistical power of 0.80, the analysis indicated that a minimum of 190 participants were required. To account for an anticipated attrition rate of 20%, the recruitment target was set at 350 participants. After excluding 42 participants due to incomplete data, a final sample of 308 participants (88%) was included in the analysis. This sample size exceeds the minimum requirement, ensuring adequate power for detecting significant effects and allowing for robust subgroup analyses.

### Physical activity measurement

Physical activity (PA) was objectively measured using the ActiGraph GT3X + accelerometer (Pensacola, FL, United States). This device has been extensively validated for measuring free-living physical activity in older adults, demonstrating high reliability and accuracy in detecting moderate-to-vigorous physical activity (MVPA). Participants were instructed to wear the accelerometer on their right hip for seven consecutive days during waking hours, except during activities such as showering or swimming.

The accelerometer data were processed using ActiLife 6 software (version 5.5) and analyzed following the algorithm developed by Troiano et al. (12). PA intensity was categorized using thresholds established by Miller et al. (8) for adults aged 60 and above: 100–1951 counts per minute (cpm) were classified as light physical activity, and activity above 1952 cpm was classified as moderate to vigorous physical activity (MVPA). Based on the World Health Organization (WHO) recommendation of at least 150 min per week of moderate aerobic physical activity, participants were classified into two groups: those meeting the guidelines (≥150 min/week of MVPA) and those not meeting the guidelines (<150 min/week of MVPA).

### Cognitive function measurement

Cognitive function was assessed using the Montreal Cognitive Assessment (MoCA), a widely used 30-point screening tool designed to assess mild cognitive impairment. The MoCA evaluates various cognitive domains, including short-term memory recall, visuospatial abilities, executive functions, attention, working memory, language fluency, and orientation to time and place. Higher scores indicate better cognitive function. The MoCA has been shown to have good sensitivity and specificity for detecting cognitive impairment in older adults (10).

### Covariates and controls

To account for potential confounding factors that could affect the relationship between physical activity and cognitive function, the following covariates were included in the analysis:

Age: participants were divided into two groups (60–70 years and 71–80 years), as previous research has shown a negative association between age and cognitive function (7).

Gender: gender was considered a key moderating variable, given the potential differences in how men and women experience changes in cognitive function and physical activity (9).

Education level: education was categorized into two groups: more than 9 years and 9 years or less of formal education (10). This cutoff was chosen based on the structure of the Chinese educational system, where 9 years of education typically corresponds to the completion of junior high school, which is the end of compulsory education in China.

Self-Rated Health Status: participants assessed their overall health by selecting one of three categories: “poor,” “general,” or “good.” While self-rated health is subjective, it has been validated as a strong predictor of objective health outcomes and mortality (11). To improve validity, self-rated health status was supplemented with objective health indicators, including the number of chronic conditions and BMI, as documented in participants’ medical records.

Body Mass Index (BMI): BMI was calculated using standard methods, and participants were classified as overweight or obese if their BMI was ≥25 kg/m^2^, based on Asian-specific standards. BMI has been linked to cognitive function (11).

Comorbidities Assessment: Comorbidities were assessed by recording the number of diagnosed chronic diseases each participant had. Participants reported any chronic conditions diagnosed by a healthcare professional, including but not limited to hypertension, diabetes, cardiovascular diseases, arthritis, and respiratory illnesses. The total number of chronic conditions was used as a covariate in subsequent analyses.

### Statistical analysis

All statistical analyses were conducted using Stata 12.0 (StataCorp, College Station, TX, United States). Descriptive statistics were used to summarize the demographic and health characteristics of the participants, and comparisons were made between participants meeting and not meeting the physical activity guidelines. Multivariable linear regression models were used to assess the association between meeting the physical activity guidelines (≥150 min/week) and cognitive function, adjusting for the aforementioned covariates. Additionally, analysis of variance (ANOVA) was conducted to explore gender differences in the relationship between physical activity and cognitive function. Interaction terms were included in regression models to assess the moderating role of gender. Effect sizes, including Cohen’s d and standardized regression coefficients, were reported to provide a clearer understanding of practical significance. All statistical tests were two-sided, and a *p*-value < 0.05 was considered statistically significant. Results were reported as regression coefficients (*β*), 95% confidence intervals (CI), *p*-values, and effect sizes where applicable.

## Results

### Descriptive statistics

Of the 308 participants included in the final analysis, 52.9% were female, and 47.1% were male. The mean age was 68.4 years (SD = 5.6), with participants divided into two age groups: 60–70 years (68.2% of the sample) and 71–80 years (31.8% of the sample). The characteristics of participants who met the physical activity guidelines (PAG) (≥150 min/week of moderate-to-vigorous physical activity) were compared to those who did not meet the guidelines (<150 min/week). Key demographic and health-related characteristics of the two groups are summarized in Table 1. Key Findings: Participants who met the PAG tended to be younger, had higher levels of education, and reported better self-rated health than those who did not meet the guidelines. Women were overrepresented in the group that did not meet the physical activity guidelines.

**Table 1:** Descriptive characteristics of the participants based on adherence to physical activity guidelines.

Characteristic	PA ≥ 150 min/week (*n* = 240)	PA < 150 min/week (*n* = 68)	*p*-value
Gender (Female)	130 (54.2%)	47 (69.1%)	0.005
Age (years)	67.2 (5.3)	70.5 (5.2)	<0.001
Education (≤9 years)	42.50%	76.50%	<0.001
Self-rated health (Good)	31.70%	41.20%	0.023
BMI (≥25 kg/m^2^)	47.90%	50.00%	0.765
Comorbidities Assessment (two or more)	22.1%	26.5%	0.836

BMI, body mass index; PA, physical activity.

### Cognitive function outcomes

The overall cognitive function scores (measured by MoCA) were significantly higher in participants who met the PAG compared to those who did not meet the guidelines. The mean MoCA score in the group that met the PAG was 25.30 (SD = 2.47), while it was significantly lower at 20.73 (SD = 2.19) in the group that did not meet the guidelines (*p* < 0.001). Effect size analysis indicated a substantial impact of physical activity on cognitive function (Cohen’s *d* = 0.85), suggesting that the observed differences are not only statistically significant but also meaningful in practical terms. Significant differences were found in all cognitive domains between the two groups. Participants meeting the physical activity guidelines had notably higher scores in executive function, memory, attention, visuospatial abilities, and language fluency compared to those who did not meet the guidelines (Table 2).

**Table 2:** Cognitive function and specific cognitive domains by adherence to physical activity guidelines.

Cognitive domain	PA ≥ 150 min/week (Mean ± SD)	PA < 150 min/week (Mean ± SD)	*p*-value
Overall MoCA score	25.30 ± 2.47	20.73 ± 2.19	<0.001
Executive function	3.51 ± 1.08	2.53 ± 0.91	<0.001
Memory	3.04 ± 1.40	1.71 ± 1.17	<0.001
Attention	2.68 ± 0.53	2.28 ± 0.59	<0.001
Visuospatial abilities	1.65 ± 0.54	1.15 ± 0.70	<0.001
Language fluency	2.05 ± 0.77	1.56 ± 0.82	<0.001

PA, physical activity; MoCA, Montreal Cognitive Assessment.

### Gender differences in cognitive function

Further analysis revealed gender-specific differences in the relationship between adherence to PAG and cognitive function. Women who met the PAG showed significantly higher cognitive function scores than men in several domains, including executive function, naming tasks, and visuospatial abilities. However, men showed better memory scores than women (*p* < 0.001 for gender interaction). The interaction effect suggests that while both genders benefit from physical activity, the cognitive domains influenced differ significantly. Women who met the PAG exhibited higher scores in executive function and visuospatial abilities than their male counterparts, whereas men showed superior memory performance. These findings suggest that the cognitive benefits of physical activity may be modulated by gender, with different cognitive domains being more sensitive to physical activity in men and women (Table 3).

**Table 3:** Gender-specific effects of physical activity adherence on cognitive domains.

Cognitive domain	Male (*β*, 95% CI)	Female (β, 95% CI)	*p*-value for interaction
Overall MoCA score	3.21 (95% CI: 2.19–4.22)	4.04 (95% CI: 3.21–4.87)	<0.001
Executive function	0.37 (95% CI: −0.09-0.83)	0.79 (95% CI: 0.43–1.16)	0.043
Memory	1.31 (95% CI: 0.64–1.97)	0.98 (95% CI: 0.52–1.43)	<0.001
Visuospatial abilities	0.25 (95% CI: 0.00–0.49)	0.47 (95% CI: 0.24–0.70)	0.017

β, regression coefficient; CI, confidence interval; MoCA, Montreal Cognitive Assessment.

### Multivariable regression analysis

The results show that the R^2^ value of the model is 0.58, indicating that factors such as physical activity, age, sex, and education explained 58% of the variance in cognitive function. After adjusting for age, education level, BMI, and self-rated health, adherence to the physical activity guidelines remained a significant predictor of cognitive function (*β* = 3.67, 95% CI: 3.04–4.29, *p* < 0.001). Age and education level also had significant effects on cognitive function, with older participants and those with less education showing lower cognitive scores. Potential estimation errors were assessed using variance inflation factors (VIFs), which confirmed the absence of significant multicollinearity in the model. Meeting physical activity guidelines had the strongest positive association with cognitive function, even after adjusting for confounding factors. Education level also played a significant role in predicting cognitive outcomes (Table 4).

**Table 4:** Multivariable linear regression of cognitive function outcomes.

Variable	*β*	95% CI	*p*-value
Adherence to PAG	3.67	3.04–4.29	<0.001
Age (71–80 vs. 60–70)	−0.85	−1.37-(−0.33)	0.001
Education (>9 years)	2.12	1.61–2.63	<0.001
BMI (≥25 kg/m^2^)	−0.58	−1.07-(−0.09)	0.021
Self-rated health (good)	0.21	−0.67-1.09	0.640

### Interaction analysis results

This study revealed a significant interaction effect between gender and physical activity on cognitive function (as measured by the MoCA score). The interaction effect was statistically significant (*p* = 0.008), indicating that men and women experience different cognitive benefits from physical activity. Specifically, the cognitive improvement associated with increased physical activity was more pronounced in men, potentially due to lower baseline cognitive levels, allowing greater room for improvement. Effect of Physical Activity Duration: Each additional minute of moderate-intensity physical activity resulted in an approximate 0.05-point increase in the MoCA score. This supports the notion that even small increments in physical activity can contribute to cognitive benefits. Gender-Specific Implications: After controlling for variables such as age and education level, men had significantly lower MoCA scores compared to women. However, physical activity had a larger impact on their cognitive improvement, particularly in memory-related tasks. Women outperformed men in executive function and visuospatial abilities, reinforcing the idea that gender is an important factor in cognitive performance (Table 5).

**Table 5:** Multivariable linear regression results for cognitive function outcomes.

Variable	Coefficient	Standard error	*t*-statistic	*P*-value
Intercept	16.6223	0.222	74.829	0.000
Physical_Activity	0.0497	0.002	25.655	0.000
Gender (male)	−1.1951	0.276	−4.329	0.000
Education (>9 years)	1.508	0.115	13.131	0.000
PA_Gender_Interaction	0.0056	0.002	2.689	0.008

## Discussion

This study provides compelling evidence that adherence to physical activity guidelines is significantly associated with improved cognitive function among older adults and highlights notable gender-specific differences in these benefits. Below, we expand upon the implications of these findings, address their potential mechanisms in more detail, and suggest directions for future research, with particular emphasis on improving both the depth and scope of our conclusions.

### Strengthening the cognitive reserve hypothesis

The findings of this study support the cognitive reserve hypothesis, which suggests that engaging in enriching activities, such as regular physical activity, can enhance brain plasticity and buffer against age-related cognitive decline (12). This hypothesis was bolstered by our use of objective accelerometer data to quantify physical activity levels, reducing recall bias often found in self-reported measures (13). Importantly, our data indicate that even moderate increases in physical activity can significantly enhance cognitive performance, particularly in executive functions and visuospatial abilities (14). Future longitudinal studies are necessary to validate whether continuous adherence to physical activity can sustain or even amplify these cognitive benefits over time (6). Such research could provide a clearer causal link between physical activity and cognitive outcomes and clarify how these relationships evolve as individuals age.

### Gender-specific cognitive benefits and underlying mechanisms

A key finding of our study was the identification of gender-specific differences in the cognitive benefits of physical activity. Women who adhered to physical activity guidelines demonstrated greater improvements in executive function and visuospatial abilities, while men showed enhanced memory performance (9). These differences may be driven by hormonal, neurobiological, and sociocultural mechanisms. Estrogen and testosterone influence brain function in distinct ways. Estrogen promotes synaptic plasticity and prefrontal cortex activity, supporting higher performance in executive and visuospatial tasks, while testosterone is associated with hippocampal functioning, potentially explaining enhanced memory outcomes in men (15, 16). Physical activity further amplifies these effects by increasing brain-derived neurotrophic factor (BDNF) expression and cerebral blood flow (17–19). These neurophysiological responses may interact with sex hormones to produce domain-specific cognitive gains. Indeed, studies suggest that women may exhibit greater prefrontal activation and men greater hippocampal engagement during cognitively demanding tasks (20).

Sociocultural factors may also play a role. Women are more likely to engage in cognitively enriching social and leisure activities, contributing to cognitive reserve (21), while men often participate in occupations requiring spatial and memory-related skills, which may reinforce memory-related functions (22). Furthermore, gender differences in physical activity patterns—where men favor structured, high-intensity exercise and women engage more in lower-intensity, longer-duration activity—may shape differential cognitive outcomes (23). To further clarify these pathways, future studies should incorporate biomarkers such as hormone levels, neuroimaging data, and inflammatory profiles (24), and consider genetic moderators such as APOE ε4 to examine individual differences in response to physical activity (25).

### Policy implications for public health interventions

Our findings have significant implications for public health strategies aimed at mitigating cognitive decline among aging populations. Given the gender-specific effects observed, targeted interventions may be more effective in optimizing cognitive benefits (26). For women, programs focusing on activities that enhance executive function and visuospatial skills, such as tai chi, yoga, and strategy-based games, could be particularly beneficial (27). For men, interventions emphasizing memory enhancement, such as aerobic exercises combined with cognitive engagement activities (e.g., dual-task training), may yield greater cognitive benefits (28). Policymakers should prioritize creating supportive environments that encourage physical activity among older adults, including developing community exercise facilities, providing subsidized fitness programs, and integrating physical activity into routine health services for the older adult (29). Gender-sensitive programs should also be specifically designed: Group-based exercise programs for women could enhance social interaction, further amplifying cognitive benefits. Technology-assisted training for men, such as virtual reality-based memory exercises, may be more engaging and effective. Additionally, public awareness campaigns highlighting the cognitive health benefits of physical activity, particularly tailored for older adults and caregivers, could increase participation rates and adherence to physical activity programs (30).

### Study limitations and future directions

Although our study contributes valuable insights, several limitations should be noted. The cross-sectional design limits causal inference, preventing us from establishing whether physical activity directly improves cognitive function or whether individuals with better cognitive function are more likely to engage in physical activity. Future longitudinal or randomized controlled trials are needed to confirm these associations (5). Another limitation is the use of accelerometers, which, while providing objective physical activity measurements, may not fully capture activities such as water-based exercises, resistance training, or activities involving upper-body movement (31). Future research should consider using complementary assessment tools, such as heart rate monitors or self-reported activity logs, to provide a more comprehensive evaluation of physical activity levels (32). Additionally, our study was conducted in a single urban Chinese population, which may limit the generalizability of our findings. Expanding research to include rural populations and other Asian or Western cohorts could help determine whether the observed gender-specific effects are consistent across different cultural and environmental contexts (33).

Future research should also explore the interaction of physical activity with other lifestyle factors, such as diet, sleep quality, and social engagement, to provide a more holistic understanding of cognitive aging (34). For example, the Mediterranean diet, known for its neuroprotective effects, may enhance the cognitive benefits of physical activity (35). Similarly, studies examining the impact of sleep duration and quality on the effectiveness of physical activity interventions may yield insights into optimizing cognitive health strategies for older adults (36).

## Conclusion

This study underscores the critical role of physical activity in promoting cognitive health among older adults and highlights the importance of gender-specific approaches. Adhering to physical activity guidelines is associated with significant improvements in executive function, memory, and visuospatial abilities, but the specific cognitive benefits differ by gender.

Practical takeaways for individuals and policymakers include: Older adults should aim for at least 150 min of moderate-intensity physical activity per week to maintain cognitive function. Gender-specific exercise recommendations should be implemented in public health initiatives. Future interventions should consider hormonal, neurobiological, and sociocultural mechanisms when designing cognitive health programs. Community-level programs and subsidized exercise facilities should be expanded to encourage participation. By integrating gender-sensitive physical activity recommendations into public health policies, we can optimize cognitive benefits for both men and women, ultimately improving quality of life and reducing the societal burden of dementia.

## Data Availability

The original contributions presented in the study are included in the article/Supplementary material, further inquiries can be directed to the corresponding author.
