# Supplementary material for: Gender differences in cognitive benefits of meeting physical activity guidelines in older Chinese adults
Source: Front Public Health. 2025 Jun 11;13:1539369. doi: 10.3389/fpubh.2025.1539369 (PMC12188982; doi:10.3389/fpubh.2025.1539369)
Supplement: Supplementary file 1 [file Data_Sheet_1.docx]

| Assessment | Testing Method | Inclusion Criteria Threshold | Exclusion Criteria Threshold | Notes |
| --- | --- | --- | --- | --- |
| Visual Acuity | Snellen Visual Acuity Test | Corrected visual acuity ≥ 0.5 (both eyes) | Corrected visual acuity < 0.5 (any eye) | Correction via glasses/contact lenses allowed |
| Hearing | Pure-Tone Audiometry (500–4000 Hz) | Air-conduction hearing threshold ≤ 40 dB (both ears) | Air-conduction hearing threshold > 40 dB (any ear) | Excludes conductive or sensorineural hearing loss |
| Cognitive Function | Mini-Mental State Examination (MMSE) | Total score ≥ 24 (≥20 for participants with ≤6 years of education) | Total score < 24 (<20 for participants with ≤6 years of education) | Assesses orientation, memory, calculation, etc. |

Supplementary Table 1. Screening Thresholds for Participant Eligibility
